# Learners’ perceptions and experiences of studying psychology online

**DOI:** 10.1007/s40692-020-00167-4

**Published:** 2020-06-13

**Authors:** Gulcan Garip, Sanju Rusara Seneviratne, Susan Iacovou

**Affiliations:** 1 University of Derby Online Learning, University of Derby, Kedleston Road, Derby, DE22 1GB UK; 2 University of Roehampton (Laureate Education B.V.), Erasmus House, Roehampton Lane, London, SW15 5PU UK

**Keywords:** Online learning, Self-regulated learning, Experiences, Interviews, COM-B model, Interpretative Phenomenological Analysis

## Abstract

This study aimed to explore the lived experiences of six international and mature online learners studying on an undergraduate psychology course to identify barriers and facilitators to studying online. A secondary aim was to deductively explore the applicability of the Capability, Opportunity, Motivation, and Behaviour model to participants' narratives related to self-regulated online learning. Online interviews with six demographically diverse participants were conducted and analysed using Interpretative Phenomenological Analysis. The overarching theme was 'the balancing act of online learners', which consisted of three major themes (and respective subthemes): (1) 'identity as an online learner' ('in today's world, we're all very busy'), (2) 'access to resources' ('importance of location' and 'comparing online to on-campus teaching and learning'), and (3) 'changing nature of social interactions' ('tutors as a crutch' and 'peer-to-peer interactions'). A number of facilitators and barriers related to these themes were identified, which are applicable to the COM-B model. The COM-B model offers a novel approach in designing and delivering learning materials and activities that may instil or help maintain self-regulated learning in online psychology students.

## Introduction

Online learning has made education accessible to those previously restricted by factors such as geographic location or employment (Allen and Seaman 2013; Christensen et al. 2011; Kumar et al. 2017; Zhu et al. 2020; Larmuseau et al. 2018). ‘Online learning’ in the context of this paper will refer to any form of learning and teaching through a primarily electronic medium; with interaction between learners and their educational materials and activities, and engagement with peers and tutors taking place synchronously or asynchronously in a virtual environment (e.g. via Blackboard, Moodle, etc.; Yanuschik et al. 2015; Mayer 2018). While the popularity of online learning can be attributed to the flexibility inherent to its medium, research has identified concerns related to student engagement (Prior et al. 2016), retention rates (Mubarak et al. 2020; Jo et al. 2015), and reported perceptions of missing out on traditional classroom experiences (Ragusa 2017; Martinez et al. 2020). Current trends indicate that a greater proportion of students engage in online learning than in the past (Li et al. 2019). This highlights the need for educators and researchers to examine practical and evidence-based models that support the development of online courses that foster self-regulated learning.

Self-regulated learning can be defined as “an activity that students do for themselves in a proactive way” (Zimmerman and Schunk 1989, p. 1) or as an “interaction” through which learning is constructed (Rhode 2009). Self-regulated learning requires the effective management of time and learning resources; self-regulated learners set goals, plan ahead, and regularly reflect on and monitor their learning process (Zimmerman 2011; Wong et al. 2019). Previous research demonstrates that self-regulated learners persevere in challenging learning situations (e.g. Cho and Kim 2013; Cochran et al. 2016; Shea and Bidjerano 2010; Li et al. 2019). Consequently, the skills and strategies associated with self-regulated learning can support successful learning in an online context (Yeh et al. 2019). Research in this field has focused on remedying poor student engagement and retention rates among online learners, with particular attention given to the self-regulated learning strategies recognised as being key correlates of student success (Wei and Chou 2020; Kahu and Nelson 2017; Tsai et al. 2013).

Several well-established models of self-regulated learning in educational psychology draw on social cognitive learning theory (Bandura 1986), self-determination, and constructivist approaches to provide a framework for developing and delivering courses (Panadero, 2017). Schraw and colleagues (2006) discuss the implications of self-regulated learning in science education, wherein self-regulated learning is conceptualised as consisting of cognition (i.e. cognitive, problem solving, and critical thinking strategies), meta-cognition (i.e. knowledge of cognition and regulation of cognition), and motivation (i.e. self-efficacy and epistemology). According to this theory of self-regulated learning, learners engage in a combination of intra-individual strategies and processes to understand and control their learning environments. While these strategies may be shaped by the learners’ interactions with others and their contexts, this view of self-regulated learning does not explicitly take the learning context into consideration.

This paper introduces an interdisciplinary and dynamic model that considers the learning context when examining the mechanisms of self-regulated learning in online psychology students (see Crutzen and Peters 2017). More recent research indicates that educators are increasingly drawing on theoretical models to increase student engagement in online courses for specific subject areas (Arvaja 2014; Garrison 2011; Hersman and Schroeder 2017; Mastel-Smith et al. 2015). Further, the present study responds to the Fifth International Summit on Information and Communication Technology in Education’s call to action to understand the learning landscape in a digital age, as it undergoes fundamental changes (Voogt and Knezek 2018). Therefore, it is imperative that we identify the experiences and expectations, as well as perceived barriers to, and facilitators of online learning that may differ in students across subject areas.

The Capability, Opportunity, Motivation, and Behaviour (COM-B) model proposed by Michie et al. (2013) posits that behaviour is influenced by the interaction of the following constructs:

- *Capability:* an individual's psychological and physical capacity to engage in the activity concerned, relies on the individual having the necessary knowledge and skills to perform the behaviour;
- *Opportunity:* all the factors that lie outside the individual that make the behaviour possible or prompt it;
- *Motivation:* all those brain processes that energise and direct behaviour, not just goals and conscious decision-making. It includes habitual processes, emotional responding, as well as analytical decision-making.

According to this model, motivation and opportunity influence an individual’s capability to generate behaviour (Michie et al. 2011). Behaviour, in the context of this study, refers to actions associated with being a self-regulated learner. As this is a dynamic model, it also explains how self-regulated learning may in turn influence the three constructs. Each psychological construct is well supported in terms of its influence on behaviour (Michie and West 2013). This is the first study to explore the application of the COM-B model in the context of self-regulated online learning in psychology. This model is unique in that an individuals’ context is taken into account in relation to the behaviour of interest. The model states that behaviour occurs when the individual perceives they are capable of carrying out the behaviour, they have the opportunity to engage in the behaviour, and are motivated to perform the behaviour. Self-regulated learners may engage in a number of behaviours that help them understand and control their online learning environments.

The model has primarily been applied to developing and understanding interventions (including online interventions) to equip people with the skills to engage in health behaviours, which rely on self-regulation. There are parallels between the skills and strategies needed to learn in the context of one’s health and to learn in a subject area (e.g. Psychology; Aunger and Curtis 2016; Wong et al. 2010), which highlights the relevance of the model in this context. We build on previously established conceptualizations of online educational courses as complex online behavioural interventions (Wong et al. 2010) to explore the utility of the COM-B model (Michie et al. 2011) when examining how behaviours associated with self-regulated learning are impacted in online psychology learners.

Shih et al. (2008) predicted that qualitative research would become a popular method for exploring student experience in an online context, due to its utility in the identification of new avenues and opportunities for research (Kember and Leung 2008; Symeonides and Childs 2015). Symeonides and Childs (2015) conducted a qualitative interview study with online learners and emphasised the need “…to recognize learners as experts of their own experience” (p. 544) to help educators identify and develop more effective ways to facilitate online learner engagement. Interpretative Phenomenological Analysis (IPA; Smith and Osborn 2008) was developed to uncover and interpret how individuals (i.e. the participants) make sense of their lived experiences, making it an ideal method to understand the perceptions and experiences of learners studying psychology online. Further, IPA—derived from phenomenology, hermeneutics, and idiography (Smith et al. 2009)—accepts personal perceptions and subjective accounts to contribute to existing theory or to generate new research questions. It also acknowledges the interpretative role of the researcher in bringing the participants’ accounts together.

### Purpose of the study

1. Taking the above together, the present study answers the following research questions that were designed to build on each other: What does online learning mean to learners studying psychology online?
2. What are the facilitators and barriers to studying online in this group?
3. To what extent is the COM-B model applicable to self-regulated learning in an online context?

The present study explored the lived experiences of online learners studying an undergraduate psychology programme at a Higher Education institution based in the United Kingdom, in accordance with the principles of IPA (Smith and Osborn 2008). These experiences were then used to identify perceived facilitators of and barriers to studying online in this group. Finally, the applicability of the COM-B model was examined to potentially identify and explain the mechanisms of self-regulated learning in this sample. This study is notable for its detailed examination of the lived experiences of online psychology students from varied backgrounds, which builds on previous research that has identified the importance of human factors when considering self-regulated learning in online platforms (Wong et al. 2018). The integration of human factors and learning theories in the development of online learning environments will facilitate more adaptive support systems that optimise learning at an individual level (Wong et al. 2018). The study is also unique in that it uses the COM-B model, which has not been used in this context previously (Keyworth et al. 2020), to interpret self-regulated learning behaviours in online psychology students. Additionally, research utilising the COM-B model is widespread in health psychology, e.g. guiding data collection and analysis in qualitative studies (Atkins et al. 2017), and informing intervention development (Barker et al. 2016). It is essential, therefore, to consider the COM-B model within the context of online learning as it addresses wide-ranging influences on self-regulated learning behavior, which in turn can support educators in improving and enhancing online learning environments (Grant et al. 2019). Beyond this, understanding and preparing students to become self-regulated learners has been made all the more relevant in these times, with the landscape of learning evolving due to the 4th industrial revolution (Hirschi 2018) and the shift towards remote learning as a result of the coronavirus pandemic (Bishop 2020).

## Methods

### Study design

The present study was designed, conducted, and analysed according to the principles of Interpretative Phenomenological Analysis (IPA; Smith and Osborn 2008), which “invite[s] participants to offer a rich, detailed, first person account of their experiences” (Smith et al. 2009, p. 56). This study utilises semi-structured interviews, which are better suited to gaining insight into previously unexplored samples. There are several examples of interview studies of online student experiences but these studies favour descriptive accounts and lack interpretation (Cochran et al. 2016). All authors were involved in the development of an interview guide containing open-ended, non-directive questions. This was an iterative process, involving the trial and revision of the questions by the study team. This process involved a consideration of what the interview was likely to cover, as well as any potential difficulties likely to arise due to the wording of the questions (Smith & Osborn, 2008). The interview questions were intentionally left open-ended to avoid restricting lines of conversation and to encourage participants to produce rich narrative accounts of their experiences studying psychology online. Box 1 presents a sample of the questions that were included in the final interview guide.

**Box 1:** Interview Guide (selected questions)

Can you please tell me why you chose to study psychology online? What were your expectations of studying online? What does ‘studying online’ mean to you? Could you describe your online experience so far? Have you experienced any opportunities while studying online? Have you faced any challenges while studying online? Could you describe your typical study habits? Could you describe your interaction with your peers online? Could you describe your interaction with your tutors online? Could you tell about how studying online may influence other life commitments?	

### Recruitment

Ethical approval for the study was granted by the host university’s Research and Ethics Committee. Participants were deemed eligible if they were an online student enrolled in an undergraduate psychology programme at a UK-based Higher Education institution, had completed at least three modules, and had been studying online for at least a year to ensure that they had sufficient experience of online learning to share. Based on the eligibility criteria, it was anticipated that participants would be well positioned to share their experiences of online learning, including their perceptions of barriers to and facilitators of online learning. An invitation to potential participants was advertised on internal course webpages. Participation was voluntary and no incentives were offered. A “relatively homogenous” (Smith et al. 2009, p. 45) sample was sought to ensure that in-depth data was gathered relating to the research questions.

### Participants

Six participants took part in a single online interview with the second author. Smith et al. (2009, p. 56) state that “there is no right answer to the question of […] sample size”, however, smaller sample sizes are dictated by the idiographic aspect of IPA. Mastel-Smith and Stanley-Hermanns (2012) recommended a sample size between 4 and 10 participants, with an addendum to adjust the sample size according to the richness of the data collected. Therefore, the sample size of 6 was considered the ideal median for our study. Given that significant insight into the lived experience of a fairly homogenous sample of students had been already reached by participant 6, we concluded that we had reached saturation of themes and decided to maintain the sample size of 6 to allow for a deeper examination of each individual case as per the tenets of IPA methodology. Table 1 presents the participants’ demographic characteristics; including interview duration, the participants’ location at the time of interview, their age, marital status, gender, nationality, employment status, and years since starting online study, as well as the number of modules completed at the time of interview. The participants were categorised as being mature students by the Higher Education Funding Council for England (2015). Informed consent was obtained from all 6 participants taking part in the study. All names were replaced with pseudonyms to protect participants’ identities during transcription by the second author.

**Table 1:** Participant demographic characteristics

Participant (duration of interview)	Nationality; current location	Age, marital status, gender	Employment	Years of study and modules completed	Psychology area of interest
1. Tae (21 min)	Vietnamese; Denmark	24, single, male	Unemployed/Volunteer	1 year of study, completed 7 modules	Organisational
2. Andrea (70 min)	Irish; Luxembourg	43, partner, female	Unemployed/Volunteer	3 years of study, 12 modules	Health
3. Jade (42 min)	Scottish; Scotland	33, single, female	Accountant (Full-time)	2 years of study, 8 modules	Counselling/Clinical
4. Karen (39 min)	Serbian; Qatar	40, married 2 children, female	Part-time employment	2 years of study, completed 4 modules	Undecided/ Educational (Potentially)
5. Sophie (30 min)	English; England	48, married 2 children, female	Catering manager (Full-time)	3 years of study, completed 10 modules	Health/Clinical
6. Edward (38 min)	English; Scotland	32, engaged, male	Part-time football coach/ full-time banker	1 years of study, completed 3 modules	Sport and Exercise

### Data collection

Participants contacted the second author and arranged an online interview (via Skype™). The participant and the second author were the only individuals present during the interview in an effort to maintain confidentiality and ensure participant comfort. Interview durations ranged between 21 and 70 min, with the average length of each interview being 40 min.

### Procedure

Interested participants contacted the second author to arrange the online interviews. Regular supervision meetings were held to review the interview process and analysis stages with the first author. All interviews were audio recorded and transcribed verbatim before being analysed in accordance with IPA.

### Data analysis

There were two stages to data analysis: (1) the iterative and inductive IPA stage to explore participants’ experiences of online learning, and (2) a deductive stage involving the mapping of the COM-B constructs against participants’ reported experiences of online learning. First, interview transcripts were read and re-read by the first and second author, and a paper trail of handwritten notes were maintained in conjunction with the initial stages of familiarisation and coding. The second stage involved inductively identifying themes by each author separately. These themes were then inputted into a spreadsheet to be organised into higher level themes. An example of this process is depicted in Table 2 (the raw analysis is available upon request from the corresponding author). Throughout this process, the first two authors met to discuss the development of themes between those initially identified, and to organise said themes into higher level categories. The third stage involved the development of a coding manual for each of the major themes and the elucidation of the inter-relationship among these themes. At this stage, the third author checked for validity and reliability of the themes by working backwards from the coding manual against the transcripts. The four broad principles presented by Lucy Yardley (2000)—sensitivity to context, commitment and rigour, transparency and coherence, and impact and importance—were used to assess the validity and reliability of the study. We have demonstrated sensitivity to context through an appreciation of the interactional nature of the IPA interview process employed, which was specifically required to show empathy and put the participant at ease (Smith et al. 2009). Further, all of the researchers were involved in the development of the interview schedule and in-depth analyses of each case to demonstrate rigour and commitment to the IPA methodology. Throughout these stages, the researchers interpreted how participants’ made sense of their experiences and perceptions of studying psychology online while acknowledging our own influence in the development of the themes presented in the paper (Smith et al. 2009). Following completion of the IPA, a deductive approach was taken by the first author to explore whether the COM-B model constructs were applicable to participant transcripts, which was then confirmed by the rest of the research team.

**Table 2:** Example of inductive coding of transcript

Participant	Raw text from transcript	Coding
Sophie	“I think doing online learning is a different way of learning. Because obviously, coz it is self-directed I think it makes you, it is making me much more um organised, I am so so much more organised than I have ever been in my life or having to be, having to be. Because I’m not only studying obviously, I’m working, and I have a family, you know I have to sort of have to juggle everything, and it’s making me organise everything”	Making sense of what it means to be an online learner among everything else in one’s life

## Results

Data analysis identified ‘the balancing act of online learners’ as being the overarching theme. This related to the participants’ experiences of balancing the following: extraneous commitments with online study (e.g. family, work, etc.), expectations arising when comparing online and on-campus teaching/learning, and lastly, social interactions with peers and lecturers online. Three major themes were identified: (i) identity as an online learner, (ii) access to resources, and (iii) the changing nature of social interactions. These three themes are interrelated and inherently linked to the overarching theme as demonstrated in the paragraphs below, and depicted in Table 3. Illustrative quotes from participants are provided to support themes.

**Table 3:** Summary table of themes

Overarching theme	Major themes	Minor themes
The balancing act of online learners	Identity as an online learner	In today’s world, we’re all very busy
	Access to resources	Importance of location Comparing online to on-campus teaching and learning
	Changing nature of social interactions	Tutors as a crutch Peer-to-peer interactions

### Identity as an online learner

A majority of the participants (i.e. Andrea, Jade, Karen, Sophie, and Tae) recognised their interest in psychology at a young age, which motivated them to pursue a psychology degree. In contrast, Edward recognised his interest in psychology due to recent life experiences as a football coach. All the participants reported their motivations for studying psychology online in order to facilitate a career change. They viewed online learning as being a means to pursue this goal in spite of the varied obstacles that might have impeded them. For most of the participants (i.e. Karen, Jade, Tae, Edward, and Sophie) studying psychology was a means to an end, whereas Andrea focused on the inherent enjoyment and satisfaction gained from studying psychology online.

To Andrea, who reported experiencing chronic pain, online learning meant the “…*freedom to be anywhere I want and be able to connect and still do study*”. The availability, accessibility, and flexibility afforded by online learning were common motivators for choosing online learning, as reported by the participants. Karen perceived online learning as being the more appropriate, though less desirable alternative to on-campus teaching and learning, at a time when she was very busy:

“...I have to say, in today’s world when we are all very busy with our own lives, its, uh, its better, it’s more appropriate… I cannot say it’s more effective, but it’s easier, in general it’s easier, when you have to organise your own time, sort of, with your family, with work, and everything else. Its more suitable, because you can, its online, you can study on your own pace, you can read your lecture on your own pace, and you can dedicate your time on the way you want, …when you study on campus, I mean, you have to follow the schedule”. (Karen)

Participants discussed the decision-making processes that were involved in the juggling of their online studies with other commitments that included work, family, social lives, and leisure activities.

“…but for me my biggest hobby, well, sort of passion is football. Ehm, and I have a season ticket holder for my local football team. That’s one thing that I’m not giving up and I refuse to give up football for studying or anything. So, on a Saturday, Saturday afternoon, my brother and I go off to football and I go home and I study. …But I think you reign your social life in a wee bit, a little bit just to, you know, you can’t have everything, when you think about your life before studying you know, you just didn't have time to study, so you have to make that time”. (Jade)

For Jade, online learning required a re-prioritisation of some activities and social encounters to make time for studying. Her account suggests that she felt the need to defend these decisions when viewed from her friends’ perspectives. Similarly, Sophie reported that online learning has resulted in her becoming more organised than she had been previously:

“…I think doing online learning is a different way of learning. Because obviously, coz it is self-directed I think it makes you, it is making me much more um organised, I am so so much more organised than I have ever been in my life or having to be, having to be. Because I’m not only studying obviously, I’m working, and I have a family, you know I have to sort of have to juggle everything, and it’s making me organise everything”. (Sophie)

When reflecting on her experience as a mature online student, Sophie concludes that she is more likely to be successful in her studies now than she would have been at a younger age:

“was a very very scary thing for me doing a degree, because obviously I’m not a young 21 year old… And I think now I’m not 18, I think its, I’m a better, I’m a better student because I’m older, and yeah, I think its been really good for me for my professional development. I’m very proud of myself actually [laugh]”. (Sophie)

Edward shared that, in his experience, approaching online learning with an open mind and a curiosity about the subject allowed him to maintain the focus he needed to ‘get the job done’ (i.e. obtain his psychology degree). Andrea echoed this sentiment to some extent, as she believed that a curious attitude towards the subject made it more rewarding to learn. Her curiosity also meant she was keen to make the most of available opportunities to learn, at the risk of scattering her attention across many different elements (i.e. modules, Massive Open Online Courses, research projects).

Jade talked about having to disguise her true motivation for studying psychology (i.e. career change to become a psychologist) to potential employers out of concern that she may not be perceived as a committed accountant:

“…I’m applying for accountancy jobs, whereas, in complete honesty, I don’t want to be an accountant in five years’ time. D’you know like, ehm, and it’s almost like that whole acting thing, so I’m going to these interviews and I’m completely being myself until they ask that question of where do you see yourself and I’m not, I don’t like lying or deceiving or that sort of thing and its. I’m very open and I tell them at the end that I am doing degree and they’ll get all interested and they ask what’s it in and whenever I say psychology, they kind of, it’s almost like first question is “why?!” D’you know and that’s. And I’m sure like, one of my, my last role actually, we ended the conversation where basically I just went, ‘as a hobby’ (laugh). D’you know like, ‘I do it in my spare time’ (laugh) but it’s not a hobby, it’s what I want my future career. But I can’t tell people that in an interview. Because they’ll look at me as not committed to being an accountant. When I, whereas I am but I don’t want to do it forever. So, that to me is quite tricky”. (Jade)

Jade’s account detailed the tension stemming from her balancing her current role as an accountant with her identity as an online psychology student in training for a career in psychology.

The authors designed the interview guide to focus solely on online learning, without encouraging participants to compare online and campus-based teaching and learning. It is therefore noteworthy that all participants, at various stages of the interview, made positive and negative comparisons between the two mediums. Tae, Edward, and Jade highlighted the importance of the online degree leading to an accredited qualification that was similar to, or the same as, what was awarded on-campus. Jade explains how the meaning of online learning has changed for her since she started studying online:

“…For me, if you’d asked me before I did this degree, ehm, online learning. I was probably one of those people that viewed online learning as not as good as attending university. Ehm, but since doing the degree, I’m seeing how much hard work it is and how much dedication it takes and I view online learning as.. same as usual, people are still working hard to do it, and I’m working hard to do it. So, I.. You know, when people say to me, oh, where do you study, I say Derby and they say, oh is it an online course, and I was like, well, yeah, but it is still the same degree. So I find myself now pushing back on people because for me I value online learning a lot more now I’m actually doing it and part of a course”. (Jade)

Karen raised concerns relating to her level of English proficiency, and worried that it was not of the same standard as her English-speaking peers. This resulted in some hesitation at contributing to discussion board activities in the virtual learning environment:

“Sometimes I have to say I do feel a bit shy, because in a group we have …[other students’] English is very proficient, in the comments it’s like ‘Wow!’ So I feel a bit shy, and I won’t do my activity, because I think ‘oh my god if I give my thoughts or express myself it will look funny,’ you know, because they sound so professional, which actually is not good, so I’m pushing myself to [do the activities]”. (Karen)

The COM-B constructs, when mapped against participants’ narratives of self-regulated learning, can be taken into consideration by educators to better facilitate self-regulated learning. The participants reported varied motivations for studying psychology online and opportunities that either facilitated or hindered how they managed their online studies. With the exception of Karen, all participants appeared confident and capable as self-regulated online learners. Karen’s concern around her English language skills clearly demonstrates her perceived lack of capability to contribute to online activities, although she recognised the detrimental impact that this was having on her online learning experience.

### Access to resources

All participants, with the exception of Edward, compared the mediums of online and on-campus teaching and learning. This tendency could have shaped participants’ expectations and experiences of online learning, and may be suggestive of a need for online education providers to establish expectations of online learning as being qualitatively different from campus-based learning. While, it is unlikely that the participants were comparing their online programmes to on-campus study on a regular basis, it was clear that using on-campus study as a reference point was useful to some participants when discussing the opportunities and challenges that arise when accessing resources online:

“...And as long as there is an internet connection, then I can do this. It means I’m not, I’m not having to worry and look at a calendar every time I want to do something. …I’m not hauling a whole load of books with me… I mean if you are on a campus, you have set term times, you’re aware that you can miss a class or two but there is always the worry about what if I miss this…” (Andrea)

Being able to access the psychology programme from different geographical locations was the most commonly identified advantage of online learning. However, for some participants, being physically removed from academic institutions, services and resources meant that disengagement from their studies when other life events took priority was perceived as being more likely than if they had been studying on-campus.

“and I guess you take more action when, if you have to go to the campus for example. Whereas, if you study online I feel, it feels very relaxing and casual when you can just stay at home in front of your computer, the same place you watch Netflix and chill, it feels very, the atmosphere feels very different, is what I mean to say”. (Tae)

While most participants focused on the ease of access to online learning materials, Karen, who was based in Qatar at the time of the interview, highlights the difficulties in accessing resources (e.g. books and journals) in developing versus developed counties. Karen expresses a fear of missing out arising from her geographic location (Qatar vs London) rather than the medium of study (online vs on-campus):

“…If you are in a big city, like in London, any actually economically developed countries, you can probably find the good libraries, but bit outside, it’s really difficult, even me living in Qatar… There is nothing when it comes to psychology, nothing. I mean there is national library, there is nothing when it comes to that particular subject…” (Karen)

This subtheme maps exclusively against the opportunity construct of the COM-B model as it focuses on factors outside of the participants and educators’ control. Taking the experiences of varied living arrangements (e.g. household, employment, location, etc.) among online learners into consideration, may help educators and providers to tailor expectations for prospective learners.

### The changing nature of social interactions

All participants reported feelings of isolation in relation to online learning as being a challenge. Despite the participants having used a variety of online mediums to communicate with others, the absence of face-to-face interaction and instant feedback appeared to be a point of tolerable apprehension in this sample:

“…sometimes I think it’s nice just to get some feedback right away as if you’re meeting them face to face. But studying online, I mean okay, there’s a forum where you can provide some questions and get some answers out of that. But sometimes, perhaps our supervisor isn’t there, isn’t available or maybe they are occupied with something else, so we don’t get the answers right away when we kind of need it. So, it could be a little bit inconvenient because of that”. (Tae)

For Edward, keeping ‘a low online presence’ was a personal choice that was necessary and allowed him to manage his other commitments. Edward talked about interacting with peers as being ‘a luxury, an added extra’ that he couldn’t afford due to his particular approach to online learning: “*I find it beneficial when I can pick things up and let it go when it suits me…My main focus is to pass the course*”. Alongside his studies, Edward balanced a full-time job at a bank, and worked part-time as a football coach.

For him, social interaction with peers and lecturers did not appear to have been perceived as being necessary to the completion of the programme: *“…I’ll use [lecturers] as a crutch, if and when I need their support*”.

Other participants had doubts about interacting with lecturers, particularly when they first started online learning. As Andrea puts it, “*I wondered about when you should contact a tutor*”. Andrea’s confidence in contacting her tutors when she had questions grew in conjunction with her familiarity with them. Further, Sophie explained how her confidence had increased over time to the point that she felt able to provide support to others; as a result she experienced increased interactions with her peers. For Jade, reading other students’ posts was daunting but as she got to know her peers through group work, she developed an appreciation for, and valued their contributions.

When considering the COM-B model, this theme includes examples related to the participants’ capability (i.e. the ability and confidence to interact with others online), opportunity (i.e. asynchronous communication modality), and motivation (i.e. reasons for or against interacting with others) to interact with peers and tutors. The social dynamics involved in online learning have the potential to either foster or impede self-regulated learning.

## Discussion

The primary aims of this study were to explore the experiences of learners studying psychology online and to identify potential barriers and facilitators to online study across the sample. The participants were mature students, three from non-UK countries, engaging in online learning in pursuit of a career change. As an increasing number of individuals opt for a career change, it is worth exploring means by which to support individuals balancing their existing careers with their studies. The present study identified the balancing act of online learners as the overarching theme, which has been overlooked in the field as research tends to focus on instructional design, learning outcomes, and student satisfaction (e.g. Symeonides and Childs 2015; Mayer 2018; Tanis 2020; Altinpulluk et al. 2019). When considering this, our findings are novel and carry implications of future value in pedagogic practice in the field of online learning.

The first theme, ‘the identity of an online learner’, reflected balancing one’s identity as an online learner with other commitments, roles, and responsibilities. This suggests that a truly student-centric approach requires that online learning practices consider the impact of learner identity (Oztok et al. 2012). It is, therefore, critical that students develop an online learner identity to facilitate self-regulated learning behaviours in their online learning experience. Students who fail to do so may find it difficult to remain engaged in online learning when faced with other commitments or distractions and may not succeed in their studies. This is supported by research conducted by Wong and colleagues (2019) that identified time management strategies and the regulation of effort as being critical to academic performance. The second theme, ‘accessing resources’, showed participants weighing the advantages and challenges of online learning. The emergence of this theme is in support of research that has established the importance of the e-learning environment in creating and maintaining positive learning attitudes, specifically an environment that considers student preferences and is adapted to specific learning situations (Wongwatkit et al. 2020; Zhu et al. 2020; Larmuseau et al. 2018). A conscious decision was made by the authors to avoid questions that specifically required that the participants compare online and on-campus study. Interestingly, all participants but one spontaneously used on-campus study as a reference point when sharing their experiences and perceptions related to online learning. Providers and educators that use campus-based learning as a reference point when presenting resources and opportunities in online learning may be contributing to unrealistic expectations of online learning and the fear of missing out on-campus-based learning (Ragusa 2017). The findings from the present study support previous research that revealed the opportunities and challenges of online learning compared to on-campus study. For example, the freedom and flexibility of online learning was viewed as a facilitator, whereas feelings of isolation and the lack of face-to-face interaction with peers and lecturers were generally seen as a disadvantage of studying online (Bernard et al. 2004).

The final theme, ‘the dynamic nature of social interactions online’, highlighted the variety of ways participants approached interactions with peers and tutors. While some participants favoured an isolated experience, others desired engagement with peers and tutors, despite reporting hesitation in doing so. Those who had interacted with peers and tutors reported increased confidence in the prospect of future interaction. Wong and colleagues (2019) highlighted the importance of these interactions in encouraging ‘help-seeking actions’. Additionally, previous research identified the ‘student–lecturer interaction’ to be critical in academic and social development in students, as well as academic achievement (Alshahrani et al. 2017; Warner et al. 2019; Ouyang et al. 2020). Online educators have a responsibility to ensure that students who are not fluent in the language of learning (in this case, English) are not hesitant at participating in discussion forums, which is described by Jenkins (2006) as the ‘participation gap’. Further, research suggests that it is important that educators reduce the fear of asking questions, and encourage help-seeking behaviours when necessary (Wong et al. 2019). While some students, such as Edward, preferred to keep a low online profile due to other commitments, encouraging students to be proactive may lead to students shifting from self-doubt when engaging with peers and tutors to taking pride in working with and helping other students, as was evident with Jade, Sophie, and Karen. The present study and previous research suggest that student-to-student mentoring may be an effective strategy for improving student retention and engagement in online learning (Boyle et al. 2010). Therefore, the provision of tools and resources that facilitate these interactions can contribute to building confidence and capability in students, motivating them to engage with other students (Prior et al. 2016), and provide opportunities for collaborative regulated learning.

The final goal of the present study was to explore whether the capability, opportunity, motivation, and behaviour (COM-B) model constructs were applicable to the experiences of the sample. The results demonstrated the utility of the COM-B model, and presents how it might be used to support online psychology students to achieve and maintain self-regulated learning. Similarly, Wong and colleagues (2019) suggested that self-regulated learning required the combination of learning motivation and strategies to affect academic performance. The COM-B constructs could guide pedagogic approaches. For example, online educators can provide students with resources and activities that facilitate and help overcome barriers to behaviours associated with self-regulated learning (e.g. asking students to identify and reflect on their motivations for studying psychology). This supports research showing that learners perform better when they are aware of their role in the learning process (Wong et al. 2019). Further, the constructs of the COM-B model can be used as a guide to develop self-assessment tools for students to identify the extent to which they demonstrate self-regulated learning strategies. Such tools and resources would benefit from being tailored to specific subject areas, and could be examined in future pedagogic research into online learning.

### Strengths and limitations

The present study successfully demonstrates the potential of COM-B model in guiding the development of strategies to facilitate mature psychology students in achieving or maintaining self-regulated online learner status. Further, this study is limited in that the sample was self-selecting, with all participants tending to be progressing well and reporting mostly positive experiences of studying online. The participants were undertaking online studies specifically to facilitate a career change; different experiences and challenges might have arisen if online students undertaking studies as part of their initial career aspirations were sampled. While the size of the sample examined in this study raises questions as to the generalizability of its findings, the nature of IPA calls for an analysis into the perceptions and experiences of a specific group within a unique setting. Further, Smith and Osborn (2015) posit that IPA research ought to be considered in terms of theoretical generalizability.

### Conclusions and implications

The present study identified several facilitators and barriers to studying psychology online. A number of experiences reported by the participants can be mapped against the COM-B constructs, suggesting that educators may be able to reinforce and highlight these experiences as contributing to developing a self-regulated learner identity. Bjork et al. (2013) conclude that self-regulated learning is a vital skill for formal and informal learning across one’s lifespan. Online educators can design teaching and learning materials that create opportunities and foster capability and motivation, as well as setting expectations and putting contingencies in place to counter factors that might hinder students’ self-regulated learning experiences. This study derives useful insight from the experiences of online psychology students from diverse backgrounds, which highlights the need for future research to explore self-regulated online learning in other subject areas. Future research could also explore students’ experiences at varied stages of their studies to identify which characteristics remain consistent and which shift over time.
